# Comparing the Rod-Like and Spherical BODIPY Nanoparticles in Cellular Imaging

**DOI:** 10.3389/fchem.2019.00765

**Published:** 2019-11-15

**Authors:** Chong Ma, Jianxu Zhang, Tao Zhang, Haojie Sun, Jing Wu, Jingwei Shi, Zhigang Xie

**Affiliations:** ^1^Department of Gastrointestinal Colorectal and Anal Surgery, China-Japan Union Hospital of Jilin University, Changchun, China; ^2^State Key Laboratory of Polymer Physics and Chemistry, Changchun Institute of Applied Chemistry, Chinese Academy of Sciences, Changchun, China; ^3^University of Chinese Academy of Sciences, Beijing, China; ^4^International Journal of Geriatrics, Jilin University, Changchun, China; ^5^Department of Clinical Laboratory, China-Japan Union Hospital of Jilin University, Changchun, China

**Keywords:** BODIPY, imaging, nanoparticles, rod, spherical

## Abstract

To design efficient nanoparticles for bioimaging, it is necessary to obtain nanoparticles with desired cellular uptake and biofunction. There are many studies have shown that cellular uptake largely depends on the geometric properties of nanoparticles. In this work, the organic nanoparticles with rod-like and spherical shapes were fabricated, and their cellular behaviors were studied and compared in detail via cellular uptake and bioimaging effect. The nanoparticles with spherical and rod-like morphology both can be internalized by HeLa and HepG2 cells, but the rod-like nanoparticles showed better imaging performance than their spherical counterpart. Above results presented that the rod-like nanoparticles possess great potential for bioimaging in efficient delivery and ideal imaging efficacy. Our studies may provide useful and fundamental information for designing efficient bioimaging systems.

## Introduction

Nanomedicines are very important for various biomedical applications because if the prolong circulation times, overcome biological barriers, and reduce system side effects (Lee et al., 2012; Kunjachan et al., 2015). More importantly, nanomedicines have the capacity to design their physicochemical parameters such as surface charge, shape, size, and surface functionalization to achieve desired properties (Biju, 2014; Aula et al., 2015). In the past decade, many studies have shown that physicochemical parameters of nanomedicines could extremely impact their functions (Zhang et al., 2015; Kinnear et al., 2017). For example, particles with diameter in 50–100 nm show a suitable size range for maximizing tumor accumulation and minimizing the ensuing clearance (Black et al., 2014). Besides the size, another important parameter is the morphology of nanomedicines (Herd et al., 2013; Blanco et al., 2015). As a matter of fact, some microorganisms with non-spherical morphologies, such as rod bacteria or polygonal adenovirus, have great capabilities to infect specific cell types (Geisbert and Jahrling, 2004). Rencently, researchers have systematically studied the different of nanomedicines with various shapes on cellular internalization (Alemdaroglu et al., 2008; Chauhan et al., 2011). Compared to their spherical counter parts, the rod-like nanoparticles have more advantages in their cellular behaviors. However, the vast majority of nanomedicines in lab studies or clinical trials are spherical because of their ease of preparation (Yan et al., 2016; Zhang et al., 2016). To date, the development of progress in morphology control has resulted in preparing kinds of non-spherical inorganic nanoparticles, and study of specific structure-function relationships between morphology and their biological behaviors had been extensively explored (Burda et al., 2005; Ni et al., 2008). Compared to the successful studies of non-spherical inorganic nanoparticles, there are few researches on the construct of non-spherical organic nanoparticles on account of the flexibility and variability of organic molecules. Moreover, it is difficult to generate organic nanostructures with the same size but different shapes. While some researches partially studied the cellular behaviors of organic nanoparticles with different shapes, more comprehensive understanding of this issue will be pivotal to design efficient, optimal nanosystems for bioimaging applications.

Among various bioimaging techniques, fluorescence imaging attracted much attention due to its numerous advantages including high sensitivity, minimal invasiveness, good temporal resolution, high contrast, and ease of use (Zhang et al., 2015; Guo et al., 2016). In particular, organic nanomaterials are advantageous for real-time cell visualizations, diagnosis, and treatment of diseases (Chen et al., 2016). While nanoparticles bioimaging indicates a interdependent role of shape, size, and surface chemistry, how to adjust the relevant parameters to obtain better imaging results is very important.

**Scheme 1:**
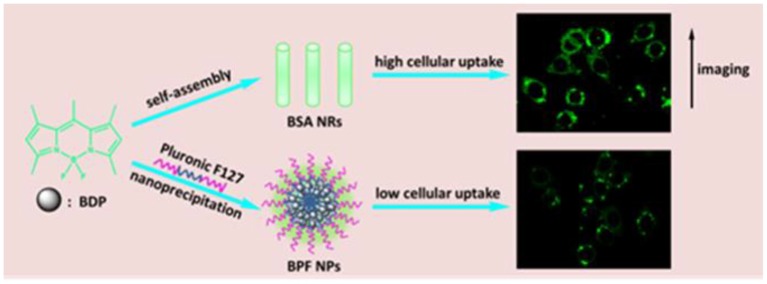
Illustration showing the preparation of spherical and rod-like nanoparticles from BDP, and comparison of cellular imaging in cancer cells.

In this paper, rod-like organic nanoparticles were synthesized by self-assembling of small molecules borondipyrromethene (BDP), and their spherical counterpart was prepared by traditional polymeric micelles. Then their ability of imaging was compared in detail (Scheme 1). BDP have attracted much attention in bioimaging because of excellent optoelectronic properties and easy of functionalization (Liu et al., 2019; Zhang T. et al., 2019). To overcome the poor water-solubility, some nanomaterials containing BDP have been prepared by physical capsulation and chemical covalent connection (Kamkaew and Burgess, 2015; Zhang W. et al., 2019). Herein, BDP was encapsulated by Pluronic F127 to acquire spherical micelles, while the corresponding rod-like nanoparticles were prepared according to our previous study. The rod-like BDP nanoparticles showed good stability and biocompatibility. Importantly, the rod-like nanoparticles showed better imaging performance than their spherical counterpart. Our studies may provide useful and fundamental information for designing efficient bioimaging systems.

## Materials and Methods

### Materials

BDP was synthesized based on the protocol reported in earlier studies. Pluronic F127 was purchased from Shanghai Yuanye Biological Technology Co., Ltd. The detailed description of the synthesis of BPF NPs and BSA NRs is shown in the Supporting Information.

### Characterization Techniques

Dynamic light scattering, transmission electron microscopy (TEM), UV-vis absorption spectra, fluorescence emission spectra, cell confocal laser microscope (CLSM), and flow cytometry were used to determine the characterization of BPF NPs and BSA NRs. Test parameters and experimental procedures are shown in the Supporting Information.

### Measurement of Fluorescence Quantum Yield

We firstly detected the concentration of BDP in BPF NPs and BSA NRs by absorbance curve, respectively. Then we adjusted them with the same concentration of BDP and also prepared BDP acetone solution with the same concentration. Fluorescence quantum efficiency was obtained on a Hamamatsu Absolute PL Quantum Yield Measurement System C9920-02.

### Cell Culture

HeLa cells and HepG2 cells were purchased from the Institute of Biochemistry and Cell Biology, Chinese Academy of Sciences, Shanghai, China. The cells were propagated to confluence in Dulbecco's modified Eagle's medium (DMEM, GIBCO) supplemented with 100 U/mL penicillin, 100 μg/mL streptomycin (Sigma) and heat-inactivated fetal bovine serum (FBS, GIBCO), and maintained at 37°C in a humidified atmosphere of 5% CO_2_ for further cell experiments.

### Biocompatibility of Pluronic F127, BDP, BPF NPs and BSA NRs *in vitro* by MTT Assay

Cells harvested in a logarithmic growth phase were seeded in 96-well plates at a density of 8 × 10^3^ cells per well and incubated in DMEM for 24 h. The medium was then replaced by 200 μL of cell culture medium within various concentrations of Pluronic F127. After incubation for 24 h, the MTT assays were used to measure the live cells. Untreated cells served as a control group. The cell survival rates (%) = A sample/A control×100%. We also detected the cytotoxicity of Pluronic F127 for different culture time. The procedures were the same for that of BDP, BPF NPs, and BSA NRs.

### Cellular Uptake and Tracking *in vitro*

The Cellular uptake of BPF NPs and BSA NRs was detected using CLSM and flow cytometry. Test parameters and detailed experimental procedures are shown in the Supporting Information.

### Studies on Endocytosis Pathway

After cells in six-well plates reaching the required density, sucrose (clathrin-mediated endocytosis, 450 mM), genistein (caveolin-dependent endocytosis, 100 μM), Amiloride (micropinocytosis, 13.3 μg mL^−1^) were used in FBS-free DMEM for 1 h and 4°C culturing was used to inhibit energy-dependent mechanisms. Then, change the culture solution within BPF NPs or BSA NRs for 4 h incubation at normal cell culture conditions. Untreated cells served as a control group. After collecting and re-suspending the cells in 0.5 mL PBS 7.4, flow cytometry analysis was performance. Test parameters and detailed experimental procedures are shown in the Supporting Information.

## Results and Discussion

### Preparation and Characterizations of BDP Nanoparticles

**Figure 1:**
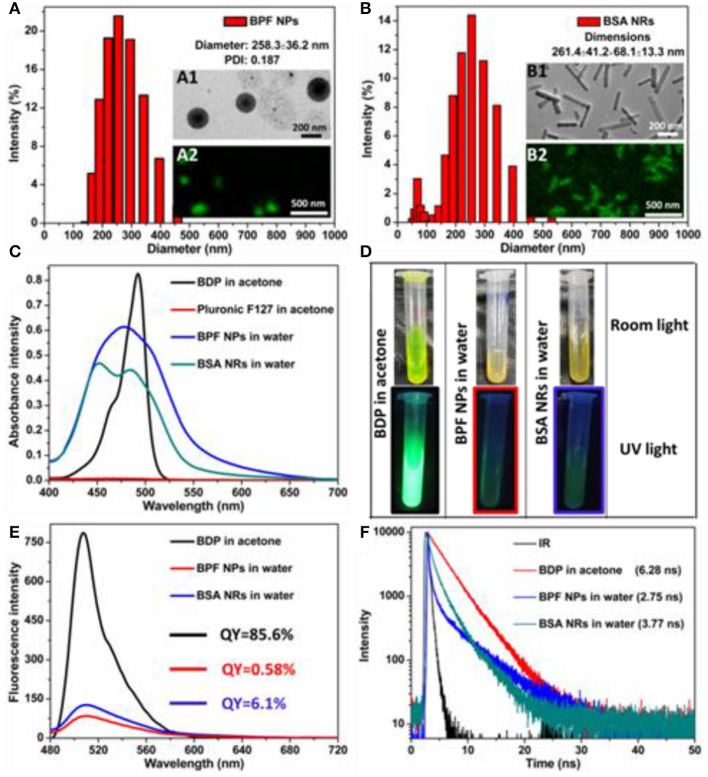
Size distribution of **(A)** BPF NPs and **(B)** BSA NRs measured by DLS in aqueous solution. Insets: TEM image and CLSM image of BPF NPs or BSA NRs. Scale bars in all TEM images are 200 nm. Scale bars in all CLSM images are 500 nm. **(C)** UV-vis absorption spectra of BDP or Pluronic F127 in acetone and BPF NPs or BSA NRs in aqueous solution, respectively. **(D)** Photos of BDP, BPF NPs, and BSA NRs in room light field and under 365 nm light irradiation, respectively. **(E)** Fluorescence spectra of BDP, BPF NPs, and BSA NRs, respectively. **(F)** Time-resolved decay profiles of BDP, BPF NPs, and BSA NRs, respectively.

According to our previous work, BDP could self-assemble into rod-like nanoparticles (BDP self-assembly, BSA NRs). Then we used Pluronic F127 to make spherical BDP nanoparticles (BDP@Pluronic F127, BPF NPs) for comparison. The size distribution and morphology of BSA NRs/BPF NPs were well-characterized by dynamic light scattering (DLS), transmission electron microscopy (TEM), and confocal fluorescence microscopy (CLSM). As shown in Figures 1A,B, the average diameters were 258.3 nm for BPF NPs and 261.4 nm for BSA NRs. TEM images revealed spherical morphology of BPF NPs (inset A1), while the BSA NRs (inset B1) was rod-like with smooth surface. These structures were further confirmed by CLSM. The BPF NPs and BSA NRs exhibited spherical and rod-like morphology with green fluorescence, respectively (inset A2 and B2). These results indicate that fluorescence BDP nanoparticles have been successfully prepared. The UV-Vis absorption of free BDP, Pluronic F127, BPF NPs, and BSA NRs were shown in Figure 1C. To compare their spectra, the concentration of BDP in all samples was same, which was adjusted according to UV-Vis standard curves (Figure S1). After the formation of nanoparticles, the UV-Vis spectra of both BPF NPs and BSA NRs showed a broad spectrum with blue-shifted absorption relative to that of BDP. Photographs of BDP in acetone, BPF NPs and BSA NRs in water (from left to right) under room light and UV light (365 nm) were shown in Figure 1D. Interestingly, compared to BDP solution, the color of BPF NPs and BSA NRs produced remarkable changes, which was consistent with spectral changes in Figure 1C. The green fluorescence of BDP was quenched after formation of BPF NPs and BSA NRs due to the typical aggregation induced quenching effect (Figures 1D,E). The quantum yield of BSA NRs was 6.1% and higher than that of BPF NPs (0.58%), which was in favor of imaging. The fluorescence lifetime (Figure 1F) of BPF NPs and BSA NRs was 2.75 and 3.77 ns, respectively, which were both shorter than that of free BDP (6.28 ns). It is well-known lifetime of fluorophores would greatly reduce when fluorescence quenching occurs. All above results further confirm that BPF NPs and BSA NRs have been successfully prepared.

### Stability of BPF NPs and BSA NRs

**Figure 2:**
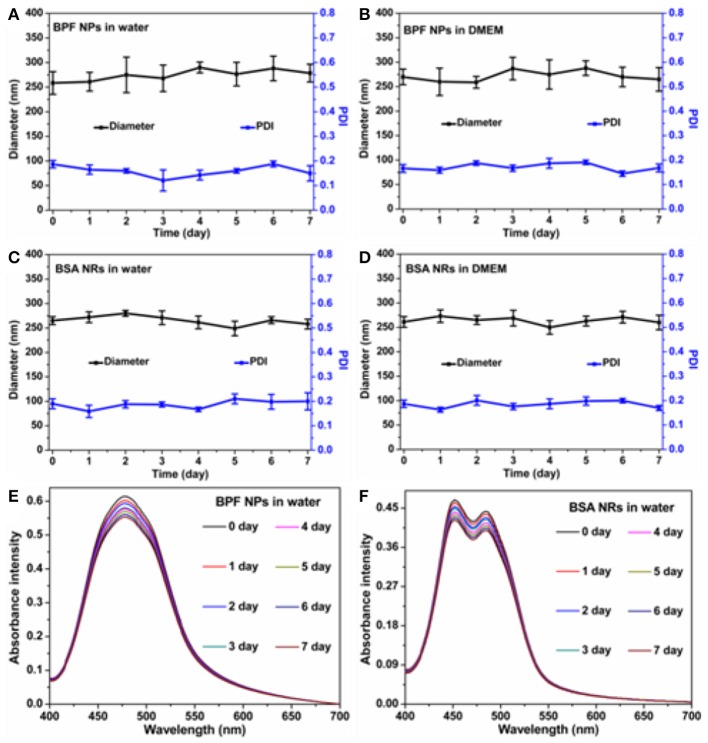
Average size changes of BPF NPs **(A,B)**, BSA NRs **(C,D)** during 7 days in different solutions. The data are shown as the mean values ± standard deviation (SD) (*n* = 3). The absorbance intensity of **(E)** BPF NPs and **(F)** BSA NRs during 7 days.

To investigate the stability of BDP nanoparticles, we monitored the size distribution and absorbance spectra of nanoparticles at various time points, and visual comparisons were made between the freshly prepared nanoparticles and the nanoparticles stored for 7 days. As shown in Figure S2, all solutions remained clear without aggregation and precipitation in 1 week. The mean sizes and size distributions of the BPF NPs (Figures 2A,B) and BSA NRs (Figures 2C,D) dispersed in aqueous solution and cell culture medium (DMEM) with 10% fetal calf serum and 1% penicillin/streptomycin changed negligibly for the whole period of time tested. Moreover, the absorbance spectra of BPF NPs and BSA NRs in water were collected during 7 days. As displayed in Figures 2E,F, the absorbance of both BPF NPs and BSA NRs declined slightly and kept more than 90% of the initial value within 7 days. These results suggest that the BPF NPs and BSA NRs possess excellent physical and optical stability, which is conducive to their application in biomedicine.

### Biocompatibility of BDP Nanoparticles

**Figure 3:**
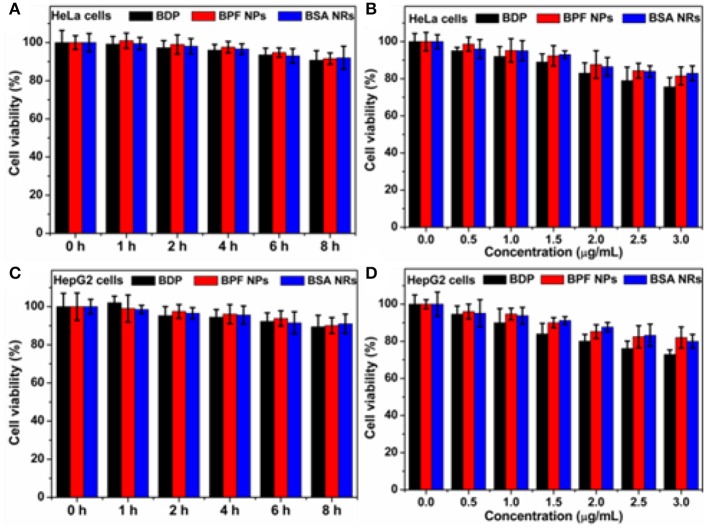
**(A)** Cell survival rate of HeLa cells after incubation with BDP, BPF NPs, and BSA NRs for different hours, respectively. **(B)** Cell survival rate of HeLa cells after incubation with various concentrations of BDP, BPF NPs, and BSA NRs for 24 h, respectively. **(C)** Cell survival rate of HepG2 cells after incubation with BDP, BPF NPs, and BSA NRs for different hours, respectively. **(D)** Cell survival rate of HepG2 cells after incubation with various concentrations of BDP, BPF NPs, and BSA NRs for 24 h, respectively. Data represent mean values ± standard deviation, *n* = 3.

The biocompatibility of nanoparticles is important for their biomedical applications. We firstly studied the biocompatibility of Pluronic F127 in living cells by the standard thiazolyblue tetrazolium bromide (MTT) proliferation test. As shown in Figure S3, little cytotoxicity of Pluronic F127 had been observed against Human cervical carcinoma (HeLa) cells and Liver hepatocellular carcinoma (HepG2), and more than 90% of those cells were alive at different incubation time or concentrations. In addition, both BPF NPs and BSA NRs showed very low cytotoxicity (90% viability) toward HeLa (Figures 3A,B) and HepG2 cells (Figures 3C,D). Therefore, the two nanoparticles are suitable for further biological research.

### Comparing Cellular Imaging Between BPF NPs and BSA NRs

CLSM was used to study the cellular uptake of BPF NPs/BSA NRs on HeLa and HepG2 cells. After incubation with BPF NPs or BSA NRs for 2 h at 37°C, the nucleus was stained by 4, 6-diamidino-2-phenylindole (DAPI). As shown in Figure S4, the bright green fluorescence appeared in the cytoplasm, suggesting that both BPF NPs and BSA NRs could be effective endocytosis by cancer cells. The intracellular fluorescence increased with the increase of BDP concentration, indicating concentration-dependent cellular uptake.

**Figure 4:**
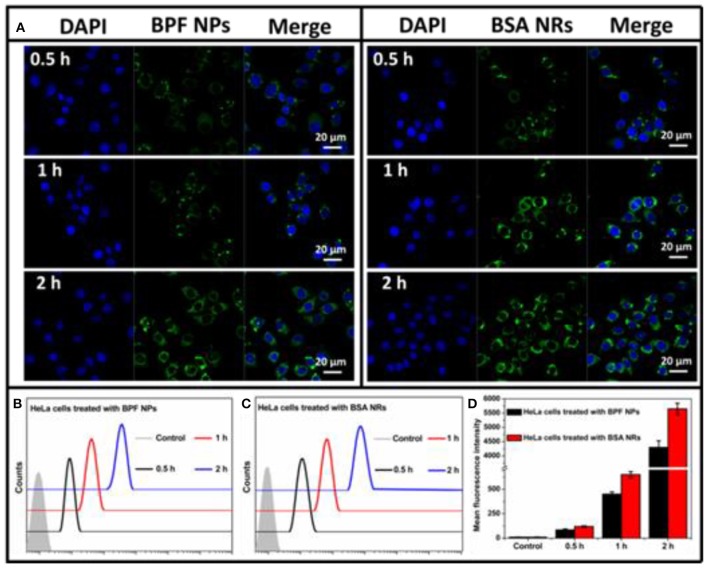
**(A)** CLSM images of HeLa cells incubated with BPF NPs or BSA NRs for 0.5, 1, and 2 h at 37°C, respectively. Cells are viewed in the blue channel for DAPI, the green channel for BDP. Scale bars represent 20 μm in all images. **(B)** Flow cytometry histograms of HeLa cells treated with BPF NPs and without treatment (control) for different hours, respectively. **(C)** Flow cytometry histograms of HeLa cells treated with BSA NRs and without treatment (control) for different hours, respectively. **(D)** Quantitative analysis of **(B,C)**. The data are presented as the mean values ± standard deviation, *n* = 3.

To further compare the cellular imaging capacity between BPF NPs and BSA NRs, the HeLa and HepG2 cells were incubated with the BPF NPs and BSA NRs (BDP: 3 μg/mL), respectively. As shown in Figure 4A, the green fluorescence intensity increased with the incubation time from 0.5 to 2 h, suggesting time-dependent endocytosis. BSA NRs exhibited stronger green fluorescence than that of BPF NPs under the same experiment conditions. Moreover, flow cytometry was carried out to further compare the cellular imaging capacity of the two BDP nanoparticles. As reported in Figures 4B,C, the endocytosis of the two nanoparticles was time-dependent, and the BSA NRs showed higher imaging efficiencies than BPF NPs (Figure 4D). These results are consistent with the CLSM results. Similar results were obtained in HepG2 cells (Figure S5). One possible trigger for higher imaging performance of BSA NRs is that rod-shaped nanoparticles have more contact sites with the cell membranes, leading to stronger adhesions and endocytosis with respect to spheres, which theoretically have only one contact point with a single cancer cell.

### Pathways of Endocytosis

**Figure 5:**
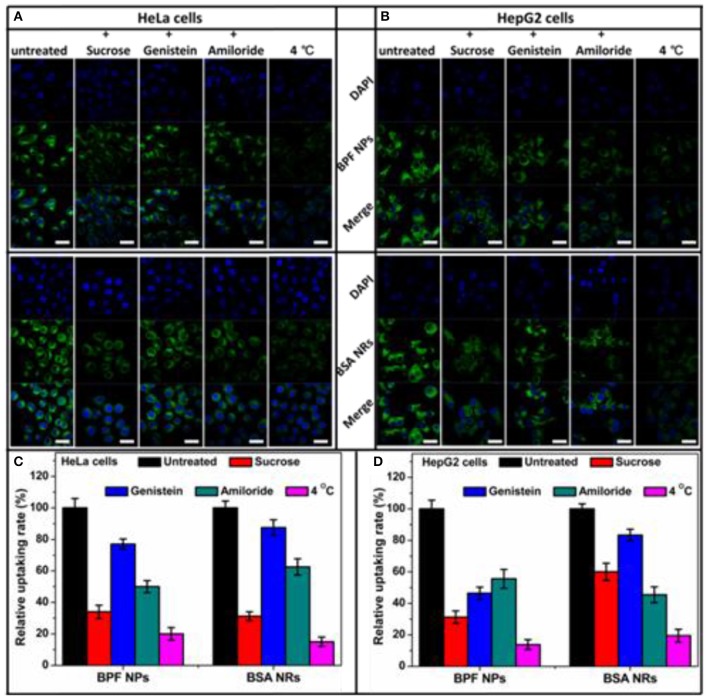
Flow cytometry histograms of **(A)** HeLa cells and **(B)** HepG2 cells treated with BPF NPs, BSA NRs and control pretreated with various endocytic inhibitors, respectively. **(C)** Quantitative analysis of **(A)**. **(D)** Quantitative analysis of **(B)**. The data are presented as the mean values ± standard deviation, *n* = 3.

To determine endocytosis pathway of BDP nanoparticles, different inhibitors were selected, sucrose for clathrin-mediated endocytosis, genistein for caveolae-mediated endocytosis, and amiloride for macropinocytosis, respectively. Moreover, the endocytosis was studied by low temperature (4°C) treatment to see if it is energy dependent. To reduce the side effects of inhibitors on cancer cells, we optimized the experiment conditions according to previous reports. The CLSM images of HeLa and HepG2 cells pretreated with inhibitors are shown in Figures 5A,B and the relative uptake rates are displayed in Figures 5C,D. The flow cytometry results are shown in Figure S6. The low temperature treatment groups all showed a sharp reduction in endocytosis of the nanoparticles, confirming endocytosis is energy dependent. The internalization of BPF NPs by HeLa cells is mainly through the clathrin-mediated endocytosis and micropinocytosis. The cellular internalization of BSA NRs through mutiple endocytosis pathways may lead to more rod-like nanoparticles internalized. Nevertheless, BSA NRs was uptaken by HepG2 cells mainly through pathway of micropinocytosis. Thus, we deduced that the endocytosis pathway varied significantly on the basis of both the physical properties of nanoparticle and the cell type, and internalization of nanoparticles with different morphologies seems to be mediated by different pathways.

## Conclusion

Herein, rod-like organic nanosystems have been rationally designed and prepared. BDP nanoparticles showed robust stability in aqueous media. *In vitro* studies have confirmed that these tailored nanosystems are biocompatible and could be uptake by living cells. Intriguingly, we found that the imaging capacity of the rod-like nanoparticles were better than their spherical nanoparticles. These results suggested a special role associated with the physical property of the particles. Our work demonstrates high-performance organic nanomaterial with ideal biological geometries for tumor imaging *in vitro*. This work highlights the potential of rational design to develop functional nanoparticles for tumor imaging.

## Data Availability Statement

All datasets generated for this study are included in the article/Supplementary Material.

## Author Contributions

CM designed the experiments, participated in all the experiments, analyzed the data, and wrote the draft of the manuscript. JZ and TZ provided assistance to the entire experimental section and contributed in the discussion. HS and JW were mainly responsible for cellular uptake and imaging experiments. ZX and JS conceived the idea and supervised the research project.

### Conflict of Interest

The authors declare that the research was conducted in the absence of any commercial or financial relationships that could be construed as a potential conflict of interest.
