# Supplementary material for: Comparing the Rod-Like and Spherical BODIPY Nanoparticles in Cellular Imaging
Source: Front Chem. 2019 Nov 15;7:765. doi: 10.3389/fchem.2019.00765 (PMC6873392; doi:10.3389/fchem.2019.00765)
Supplement: Supplementary file 1 [file Data_Sheet_1.docx]

**Electronic supplementary information**

**Comparing the Rod-like and spherical BODIPY nanoparticles in cellular imaging**

**Chong Ma^1^, Jianxu Zhang^2,3^, Tao Zhang^1^,Haojie Sun^1^ , Jing Wu^5^, Jingwei Shi^4^*, Zhigang Xie^2^,***

1. Department of Gastrointestinal Colorectal and Anal Surgery, China-Japan Union Hospital of Jilin University, 126 Xiantai Street, Changchun, Jilin 130033, P. R. China.

1. State Key Laboratory of Polymer Physics and Chemistry, Changchun Institute of Applied Chemistry, Chinese Academy of Sciences. 5625 Renmin Street, Changchun, Jilin 130022, P. R. China.

1. University of Chinese Academy of Sciences, Beijing 100049, P. R. China.
2. Department of Clinical Laboratory, China-Japan Union Hospital of Jilin University, 126 Xiantai Street, Changchun, Jilin 130033, P. R. China. E-mail: shi123jingwei@163.com
3. International Journal of Geriatrics, Jilin University, Changchun, Jilin130000, P.R. P. R. China.
   - Correspondence: [shi123jingwei@163.com](mailto:shi123jingwei@163.com) (Jingwei Shi); [xiez@ciac.ac.cn (Zhigang](mailto:xiez@ciac.ac.cn%20(Zhigang) Xie)

**Materials and methods**

*Materials.*

Pluronic F127 was purchased from Shanghai Yuanye Biological Technology Co., Ltd. MTT (3-[4,5-dimethylthiazol-2-yl]-2,5-diphenyltetrazoliumbromide) was purchased from Shanghai Beyotime Biotechnology Co., Ltd. All of the other Chemicals and reagents were acquired from commercial sources without further purification, unless otherwise noted. All the solvents were purified according to the standard methods whenever needed. Milli-Q water was collected from a Milli-Q system (Millipore, USA).

*Characterization Techniques*

Diameter and diameter distribution of the nanoparticles were determined by Malvern Zeta-sizer Nano for dynamic light scattering (DLS). The measurement was carried out at 25 ^o^C and the scattering angle was fixed at 90^o^. Transmission electron microscopy (TEM) images were taken by a JEOL JEM-1011 (Japan) at the accelerating voltage of 100 kV. To prepare specimens for TEM, a drop of NPs solution (0.1 mg mL^-1^) was deposited onto a copper grid with a carbon coating. The specimens were air-dried and measured at room temperature. UV−vis absorption spectra were recorded via a Shimadzu UV-2450 UV−vis scanning spectrophotometer. Fluorescence emission spectra were conducted on a LS-55 fluorophotometer. Fluorescence quantum efficiency was obtained on a Hamamatsu Absolute PL Quantum Yield Measurement System C9920-02. The cell confocal images were obtained using Zeiss confocal laser microscope (ZEISS LSM 700). Flow cytometry was carried out on Guava easyCyte 6-2L Base System (Merck Millipore, USA).

*Synthesis of Nanoparticles*

BSA NRs were prepared using a reprecipitation method. In a typical procedure, the BDP solution (400 μL) was quickly dropwise dispersed into 4 mL of milli-Q water with vigorous stirring at room temperature for 30 min. Then the solution was dialyzed against Milli-Q water for 24 h, the cutoff molecular weight of the dialysis bags is 3500.

BPF NPs were prepared using Pluronic F127 to assemble BDP in water. In a typical procedure, firstly, the Pluronic F127 solution (200 μL) was mixed with the BDP solution (400 μL), then the mixing solution was added dropwise to the 4 mL of milli-Q water with vigorous stirring at room temperature for 30 min. Then the solution was dialyzed against Milli-Q water for 24 h, the cutoff molecular weight of the dialysis bags is 3500.

*Cellular uptake and tracking in vitro.*

The Cellular uptake of nanoparticles was examined by using a confocal laser scanning microscope (CLSM). Cells harvested in a logarithmic growth phase were seeded in 6-well plates (a sterile cover slip was put in each well) at a density of 2.5×10^5^ cells/well and incubated in DMEM for 24 h. The medium was then replaced by 2 mL of DMEM containing nanoparticles and incubated for different hours at 37°C, and further washed using PBS for 3 times.

For the CLSM detection, the cells were fixed with 4% of paraformaldehyde solution for 10 min. After that, DAPI (4,6-diamidino-2-phenylindole) was added for another 5 min incubation to locate the nucleus. Later, the cells were washed with PBS and observed using confocal laser scanning microscopy (CLSM, Zeiss LSM 700).

For the flow cytometry detection, the cells were washed with PBS and treated with trypsin. The harvested cells were suspended in PBS and centrifuged at 1000 rpm for 5 min. The supernatants were discarded and the cells were washed again with PBS to remove the medium. After washing, the cells were re-suspended in 500 μL PBS before analysis by Guava easyCyte 6-2L Base System (Merck Millipore, USA).

*Endocytosis pathway detection.*

Cells harvested in a logarithmic growth phase were seeded in 6-well plates at a density of 2.5×10^5^ cells/well and incubated in DMEM for 24 h. Flow cytometry analysis was performed by Guava easyCyte 6-2L Base System (Merck Millipore, USA) which collected 1×10^4^ gated events for each sample.

**Figures**


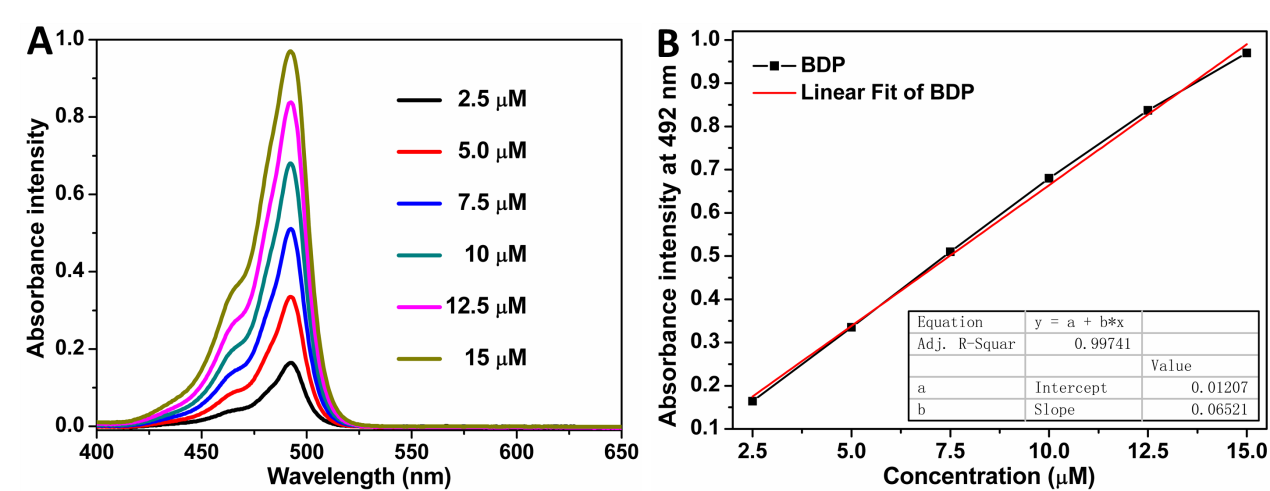


**Figure S1.** A) and B) Standard absorbance curve of BDP. (The absorbance of BDP molecules at 492 nm (from a mixture of acetone and water (v/v = 4:1)) as a function of BDP concentration.


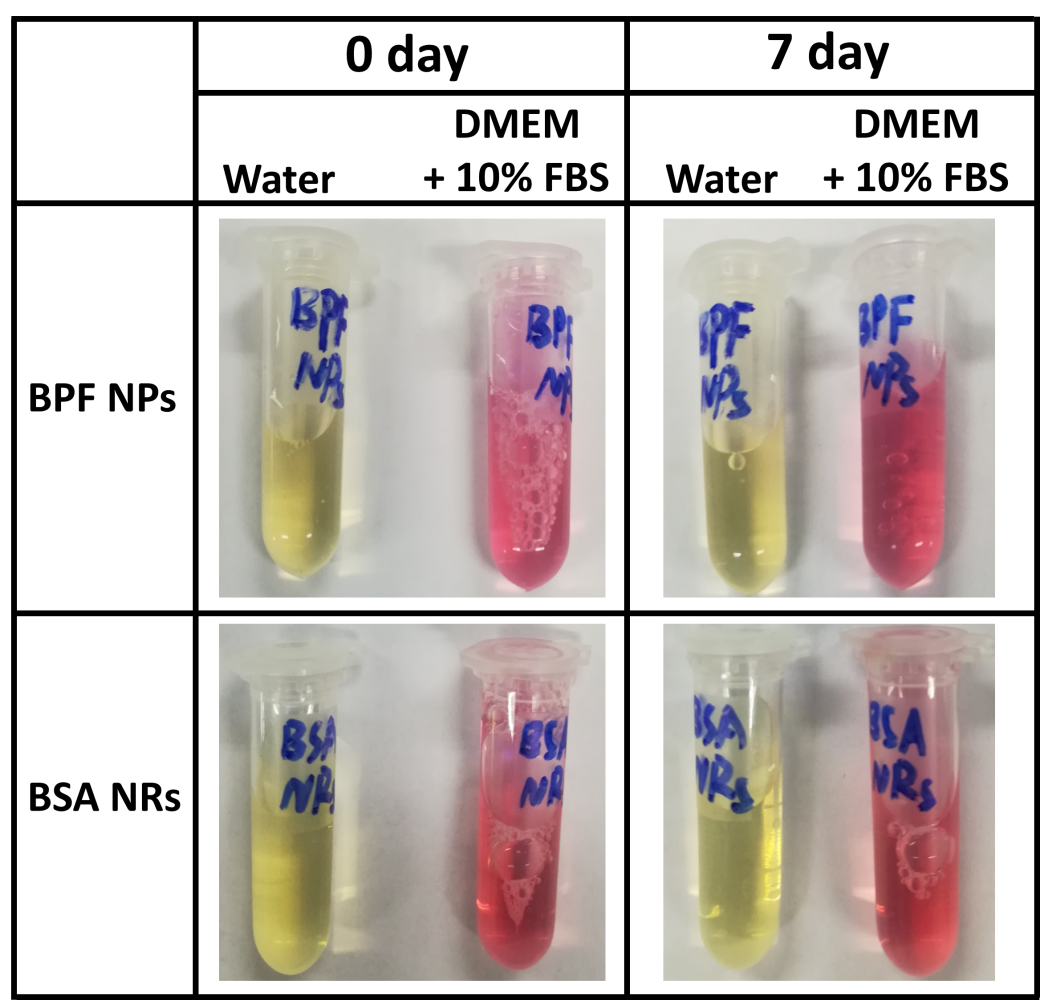


**Figure S2.** Photographs of BPF NPs and BSA NRs under different conditions at 0 day and 7 day, respectively.


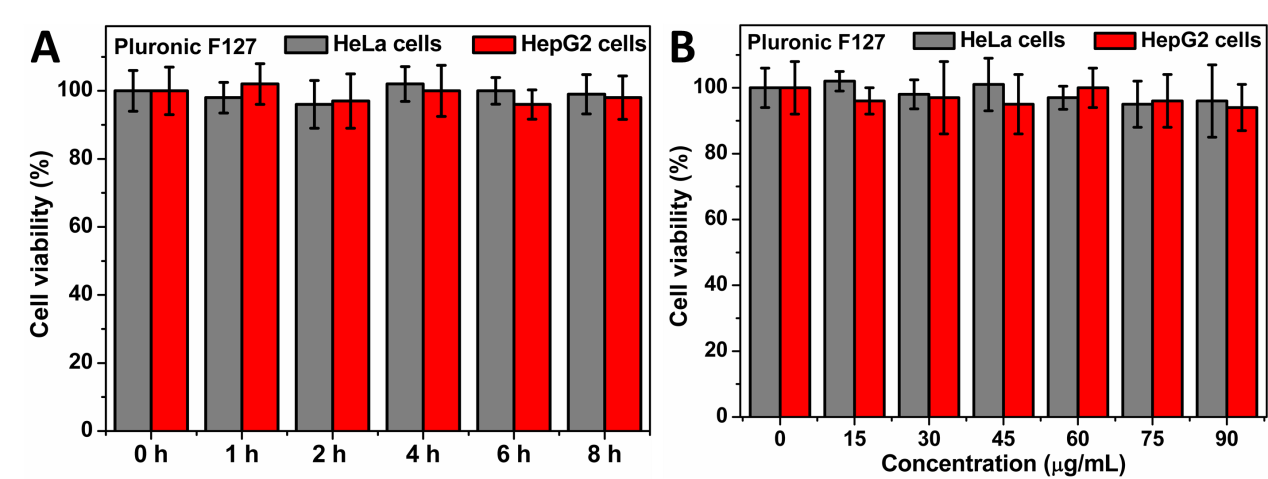


**Figure S3.** A) Relative cell viabilities of HeLa cells and HepG2 cells incubated with 90 μg/mL of Pluronic F127 for different hours, respectively. B) Relative cell viabilities of HeLa cells and HepG2 cells incubated with different concentrations of Pluronic F127 for 24 h, respectively.


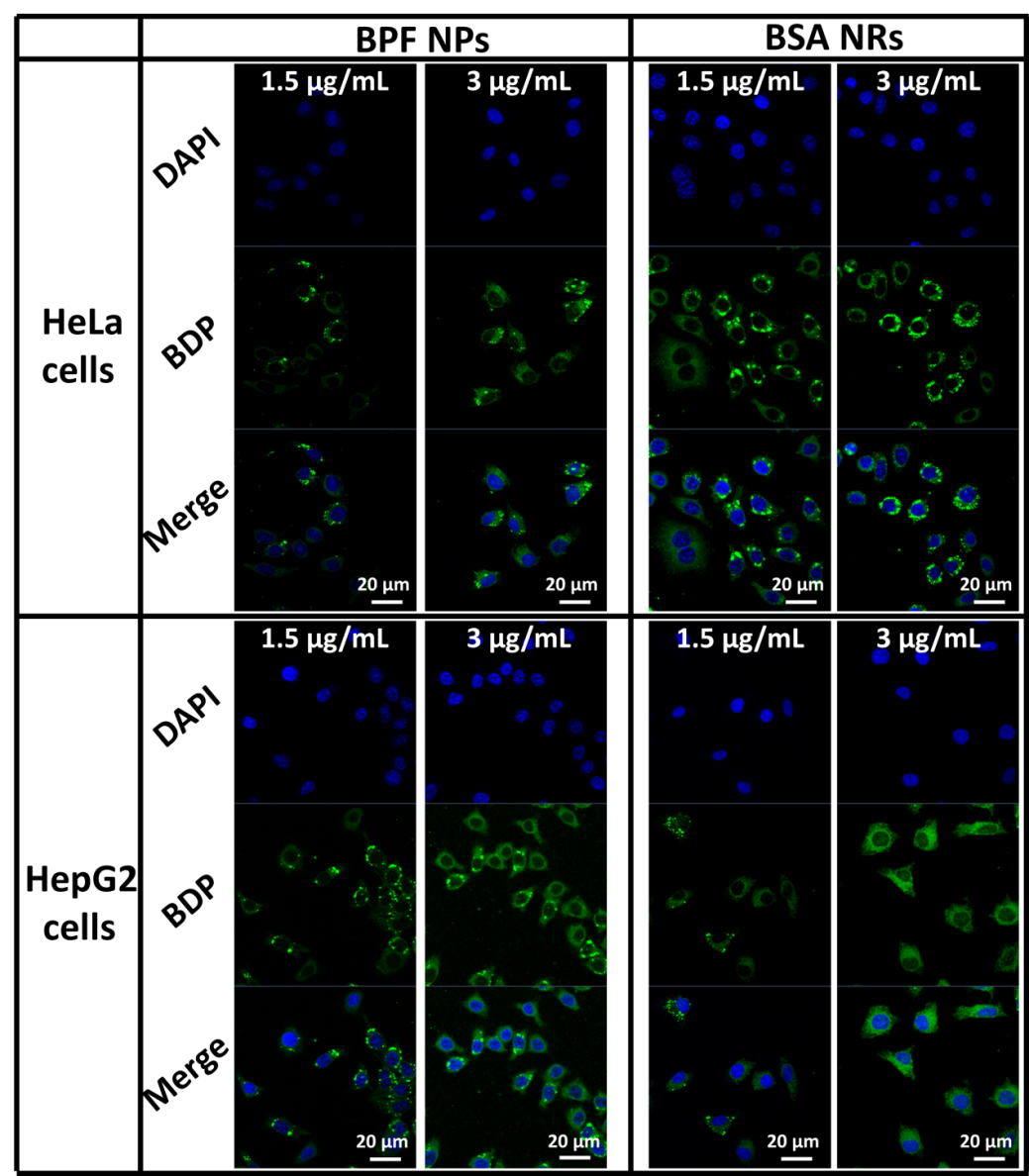


**Figure S4.** CLSM images of the cellular uptake of BPF NPs and BSA NRs in HeLa cells (upper pictures) and HepG2 cells (lower pictures) with different concentration of BDP at 2 h, respectively.


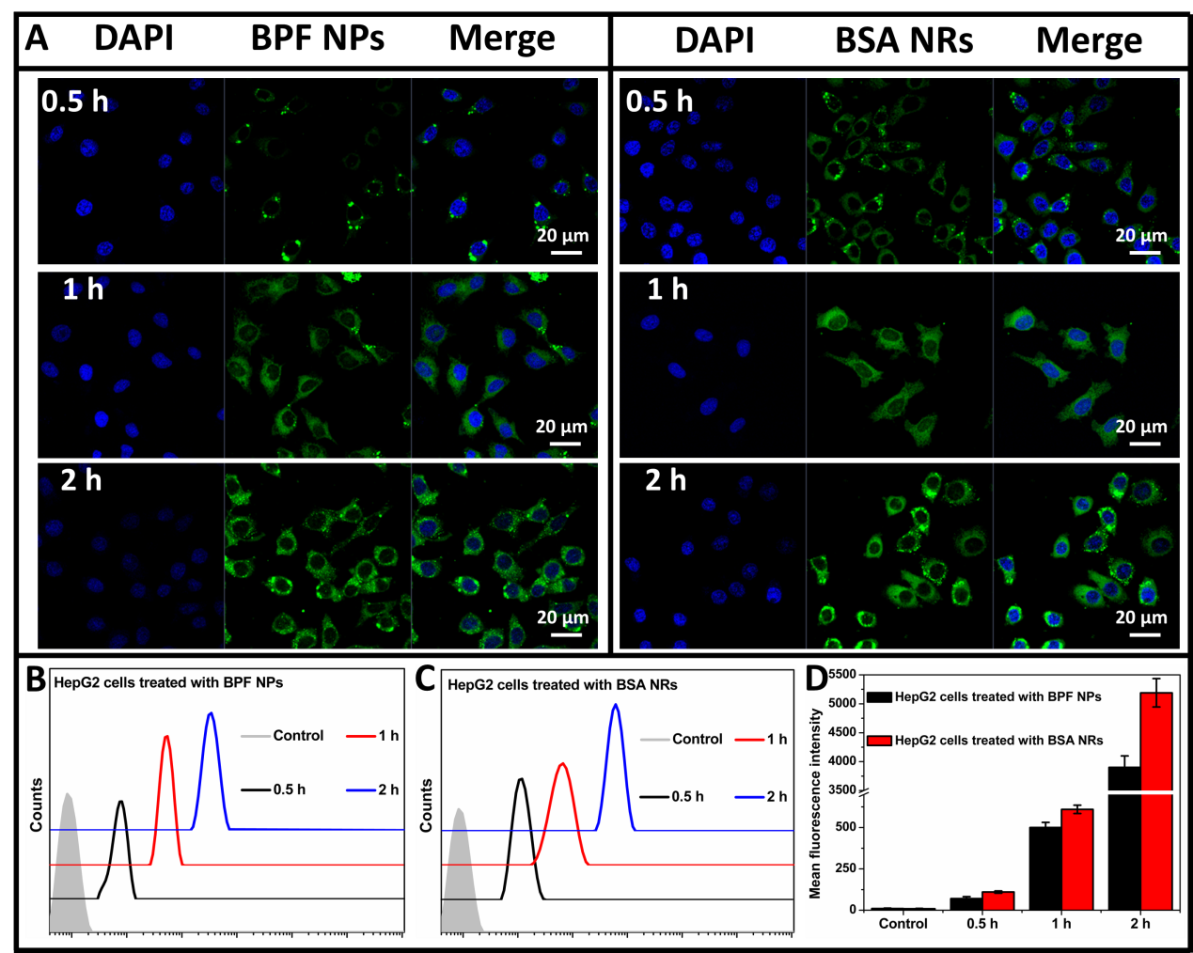


**Figure S5.** A) CLSM images of HepG2 cells incubated with BPF NPs or BSA NRs for 0.5 h, 1 h and 2 h at 37^o^C, respectively. Cells are viewed in the blue channel for DAPI, the green channel for BDP. Scale bars represent 20 μm in all images. B) Flow cytometry histograms of HepG2 cells treated with BPF NPs and without treatment (control) for different hours, respectively. C) Flow cytometry histograms of HepG2 cells treated with BSA NRs and without treatment (control) for different hours, respectively. D) Quantitative analysis of B) and C). The data are presented as the mean values ± standard deviation, n=3.


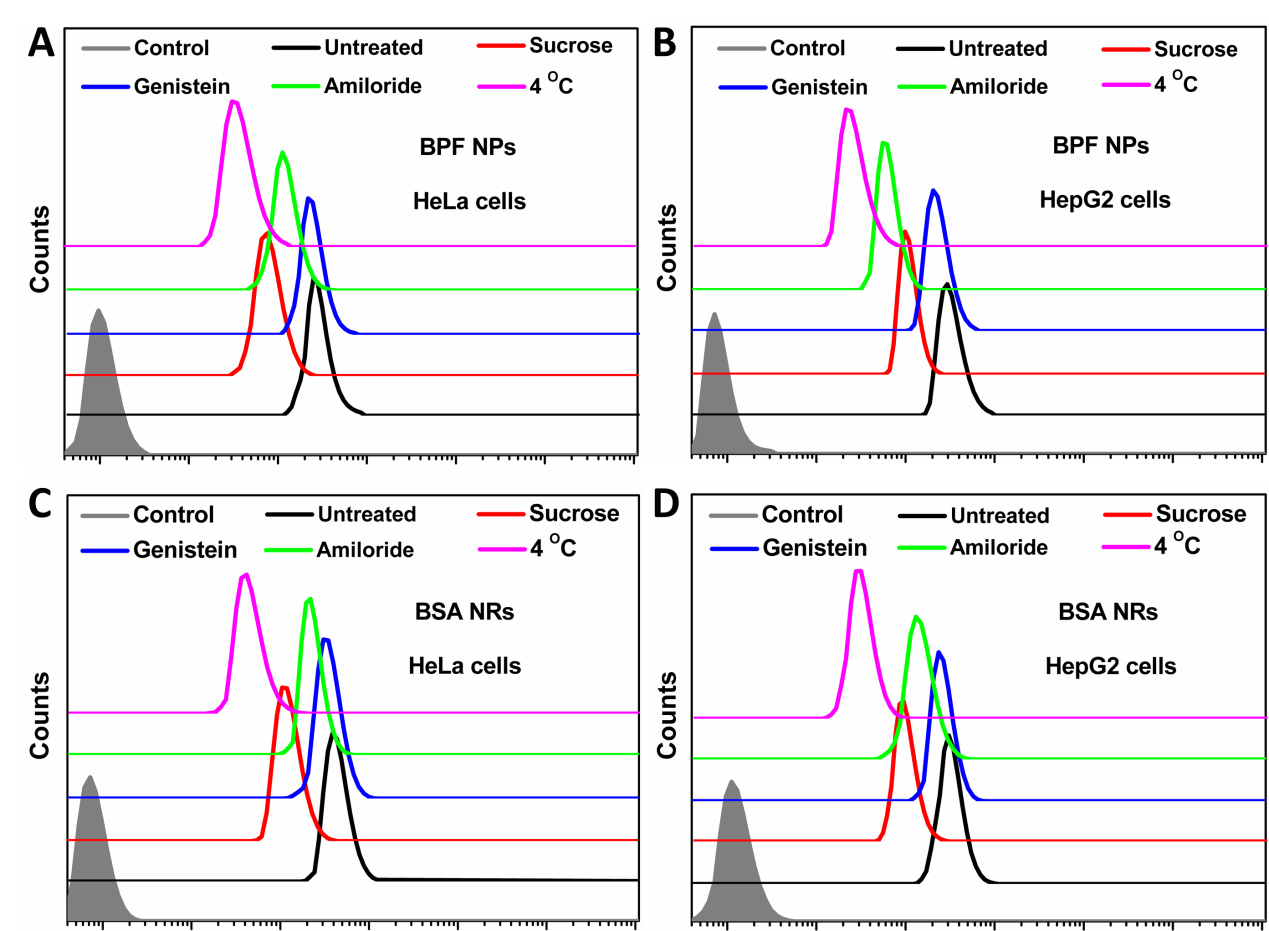


**Figure S6**. A-D) Flow cytometry histograms of HeLa cells or HepG2 cells treated with BPF NPs, BSA NRs and control after treated with different endocytic inhibitors, respectively.
